# Enhancing noncommunicable public health programs: A system dynamics approach to understanding reach, maintenance, and implementation costs

**DOI:** 10.1007/s00103-025-04069-7

**Published:** 2025-05-30

**Authors:** Simon Keith Chiu, Alix Hall, Louise Freebairn

**Affiliations:** 1 Hunter Medical Research Institute, The Population Health Research Program, New Lambton Heights, New South Wales Australia; 2 Faculty of Health and Medicine, University of Newcastle, School of Medicine and Public Health, Callaghan, New South Wales Australia; 3 Sax Institute, The Australian Prevention Partnership Centre, Sydney, New South Wales Australia; 4 Hunter New England Local Health District, Hunter New England Population Health, Wallsend, New South Wales Australia; 5 Australian National University, National Centre for Epidemiology and Population Health, Canberra, Australian Capital Territory Australia; 6 Booth Building, Wallsend Health Services, Hunter New England Population Health, Longworth Avenue, 2287 Wallsend, NSW Australia

**Keywords:** Health prevention, Sustainability, RE-AIM framework, System dynamics, Implementation, Gesundheitliche Prävention, Nachhaltigkeit, RE-AIM-Framework, Systemdynamik, Implementierung

## Abstract

**Introduction:**

Noncommunicable diseases (NCDs) cause an estimated 41 million deaths globally each year, with cardiovascular diseases, cancers, chronic respiratory diseases, and diabetes being the leading contributors. Modifiable risk factors elevate the risk of NCDs. Despite the availability of effective prevention programs, their consistent implementation remains limited, highlighting the need for strategies to improve delivery and reduce costs. The reach, efficacy, adoption, implementation, and maintenance (RE-AIM) framework and system modelling can help in understanding and improvement of the implementation and maintenance of public health initiatives.

**Methods:**

This research used a system dynamics model to explore hypothetical pathways between reach, maintenance, and the cost-related components of implementation within the RE-AIM framework. Scenarios explored the influence of strategies for enhancing reach and maintenance and the resulting impact on participation and cost.

**Results:**

Scenarios that considered alternative pathways to engagement (i.e. enhanced reach strategies) and re-engagement led to higher participation rates by utilising alternative pathways and reducing dependence on initial reach. However, this increased overall program costs due to the population cycling between engagement and disengagement.

**Conclusion:**

This modelling study highlights the complex non-linear relationships that affect the implementation of public health prevention, emphasising the need for support strategies to maintain engagement. The study shows that while alternative pathways to reach can improve participation, disengagement remains a significant challenge, impacting overall costs. Sustainment strategies that are both effective and cost-efficient are crucial for maintaining engagement and enhancing the effectiveness of public health programs.

**Supplementary Information:**

The online version of this article (10.1007/s00103-025-04069-7) contains supplementary material, which is available to authorized users.

## Abstract (de)

**Einleitung:**

Nichtübertragbare Krankheiten (NCDs) verursachen weltweit jährlich schätzungsweise 41 Mio. Todesfälle. Herz-Kreislauf-Erkrankungen, Krebs, chronische Atemwegserkrankungen und Diabetes sind dabei die Hauptursachen. Trotz der Verfügbarkeit wirksamer Präventionsprogramme ist deren konsequente Umsetzung begrenzt. Dies unterstreicht den Bedarf an Strategien zur Verbesserung der Umsetzung und Kostensenkung. Das RE-AIM-Framework („reach, efficacy, adoption, implementation and maintenance“) sowie die Systemmodellierung können dazu beitragen, die Umsetzung und Aufrechterhaltung von Programmen im Bereich der öffentlichen Gesundheit zu verstehen und zu verbessern.

**Methoden:**

In dieser Studie wurde ein Systemdynamikmodell verwendet, um hypothetische Pfade zwischen Reichweite, Aufrechterhaltung und den kostenbezogenen Komponenten der Umsetzung von Gesundheitsprogrammen anhand des RE-AIM-Frameworks zu untersuchen. In verschiedenen Szenarien wurden der Einfluss von Strategien zur Verbesserung der Reichweite und Aufrechterhaltung von Gesundheitsprogrammen sowie die daraus resultierenden Auswirkungen auf Teilnahme und Kosten untersucht.

**Ergebnisse:**

Szenarien, die alternative Wege zur Einbindung (d. h. Strategien zur Erhöhung der Reichweite) und zur Wiedereinbindung in Betracht zogen, führten zu höheren Beteiligungsquoten, indem die Abhängigkeit von der anfänglichen Reichweite verringert wurde. Dies erhöhte jedoch die Gesamtkosten des Programms, da die Bevölkerung zwischen Einbindung und Loslösung schwankt.

**Schlussfolgerung:**

Diese Modellstudie hebt die komplexen, nichtlinearen Beziehungen hervor, die sich auf die Umsetzung der Prävention im Bereich der öffentlichen Gesundheit auswirken, und betont die Notwendigkeit von Unterstützungsstrategien zur Aufrechterhaltung des Engagements und der Beteiligung. Die Studie zeigt, dass alternative Wege zwar die Teilnahme verbessern können, die Loslösung von Programmen jedoch weiterhin eine große Herausforderung darstellt, die sich auf die Gesamtkosten auswirkt. Erhaltungsstrategien, die kosteneffizient und wirksam sind, sind von entscheidender Bedeutung, um das Engagement aufrechtzuerhalten und die Wirksamkeit von Programmen im Bereich der öffentlichen Gesundheit zu verbessern.

## Introduction

Noncommunicable diseases (NCDs), also known as chronic diseases, are responsible for 41 million deaths globally each year. Cardiovascular diseases, cancers, chronic respiratory diseases, and diabetes are the leading causes of these deaths [1]. The main modifiable risk factors contributing to NCDs include tobacco use, physical inactivity, unhealthy diets, and the harmful use of alcohol. These behaviours significantly increase the risk of developing chronic diseases [1]. The economic burden of NCDs includes significant costs to the health system, individuals, and their families. Effectively applying preventive policies and practices can address up to 50% of all known health risk factors [2]. Despite the availability of effective health initiatives, their widespread adoption is uncommon [3]. Implementation science can help to inform strategies to carry out these policies and put initiatives into practice [4].

Public health prevention programs can be complex and challenging to implement at scale, with evidence often showing a reduction in the health benefits when scaled up compared to those observed in efficacy trials [5]. The conditions that lead to a prevention program’s effectiveness in one local context may not be applicable when delivered at scale to a wider community. Studying local system dynamics and the knowledge generated from practitioners can reveal the circumstances that facilitate program efficacy in different contexts. This understanding is vital for supporting the sustained implementation of a public health prevention program [3]. The RE-AIM framework (reach, efficacy, adoption, implementation, and maintenance) is one framework for considering the translation of evidence into practice. This is a planning and evaluation framework comprising five domains that underpin evidence translation and contribute to public health impact: reach—percentage and representativeness of the target population who receive the benefits of the program; effectiveness—the impact of the program protocol on important outcomes; adoption—proportion and representativeness of the setting and those delivering the program; implementation—the cost of the program and the extent to which it is delivered as intended; and maintenance—the extent to which the behaviour is sustained long term [6]. In a recent extension of RE-AIM, the importance of long-term sustained delivery and behaviour change in achieving public health impact was highlighted. Further, this extension raised awareness that RE-AIM is a dynamic rather than static process in which the domains are interrelated and influenced by each other, as well as by the application context [7].

The RE-AIM framework was developed to be compatible with systems-based and social-ecological thinking [6]. As such, these concepts can occur at an individual level and at a collective or organisational level. Reach, effectiveness, and maintenance are characteristics of individual participants of a targeted population that a public health program aims to benefit [6]. Additionally, adoption, implementation, and maintenance are focused on the uptake and delivery of the program by relevant settings and staff and thus operate at the organisational level [6]. To generate population-level impact, the target population must extend access to and engage with an intervention, which requires successful adoption and continued delivery by relevant organisations.

Public health implementation mirrors many of the features of complex systems, exhibiting non-linear behaviour and feedback loops, having numerous interacting components, and being resistant to central control [8]. These features can make it difficult to anticipate how changes between components will impact overall system behaviour and outcomes [9]. Understanding the implications of proposed policies in public health is a crucial area of research that can support evidence-based decision-making and maximise the use of limited government resources [10].

The development of generalised theories and frameworks (such as RE-AIM) can enhance the implementation of sustainability in public health programs by improving the understanding of the complex factors that contribute to why some programs are successful [11]. Simulation modelling studies can complement theoretical frameworks by examining dynamic interactions between scale-up and sustainability factors in public health programs [12, 13]. Models can help develop and test hypotheses about frameworks, exploring how system behaviour emerges and enhancing the insights gained from these frameworks [14]. Research into model structure and their insights can then support policy design and assess the implications of alternative options [15].

One such simulation modelling approach is system dynamics (SD) modelling, a computational–mathematical method that reflects components of real-world systems using differential equations to simulate changes over time [16]. Models draw on system science to facilitate a better understanding of problems and their responses to different actions, enabling decision-makers to identify more effective decision strategies [15, 17]. Through scenario analysis, changes in model input conditions represent the implementation of policy and its impact on outcomes [18].

The SD modelling methodology has useful characteristics that allow it to describe and communicate complex systems. The models are commonly coded in graphical syntax, making the model structure more accessible and providing greater transparency [19]. Data from multiple sources, including hypothetical or expert opinions, can be used to explore system behaviours [16]. In addition, SD models have relatively low computing requirements, allowing modellers to explore a wide range of scenarios or input conditions efficiently [19, 20]. These methods have been used to model a range of public health issues, including disease epidemiology, communicable diseases [21], chronic diseases [22, 23], substance abuse [24], and health care service planning [25].

This study uses SD modelling with hypothetical data to simulate the pathways in which cohorts of individual participants move through a public health program and to consider how changes in pathways impact participation and implementation costs using the RE-AIM framework. The two main study objectives are to (1) provide proof of concept of how systems modelling can be used to explore the dynamic process of the RE-AIM framework and (2) to conduct a scenario analysis to explore the interconnected nature of the reach and maintenance domains of RE-AIM and the impact of implementation costs.

## Methods

### Model structure

An SD model structure was developed to reflect the implementation components process through the RE-AIM framework’s lens. We use scenario-based modelling to demonstrate the dynamic and complex nature of implementation. Changes in the organisational domains of RE-AIM, including adoption, implementation, and maintenance—represented here by varying levels of implementation and sustainment support strategies—affect the public health impact of a program through their influence on the individual domains of reach and maintenance, represented by exposure, participation, disengagement, and re-engagement with the public health program.

**Fig. 1:**
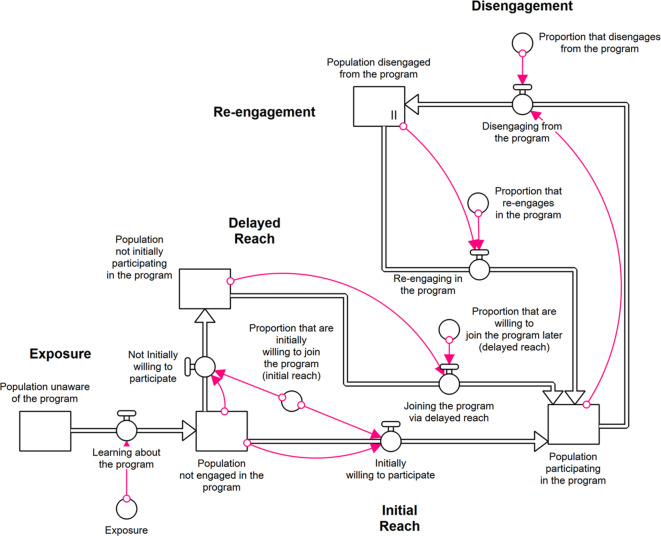
Model structure showing the pathway through phases of exposure, reach, and participation

Figure 1 depicts the modelled pathway through which the population progresses during the implementation of the health program. Before the program’s rollout, the population is initially unaware of the program. As awareness of the program increases through exposure, a proportion of the population will initially participate or remain unengaged, with a chance to become involved later through the delayed-reach pathway. Once participants actively engage in the program, they can disengage from the program, with additional pathways included in the model to allow for re-engagement. The cycle of engagement and disengagement reflects the program’s maintenance of effect. The magnitude of model flows between each state of engagement are altered to test different scenarios.

Table 1 summarises the definitions of each RE-AIM dimension and how they are reflected in the model structure. Reach is the primary outcome of the model and is measured as the proportion of the population actively participating in the program. Maintenance is represented by allowing participants to disengage from active participation in the health program. For simplicity, we assume that the program has established effectiveness and implementation fidelity that does not change over time. The organisational-level domains of RE-AIM (i.e., adoption, implementation, and maintenance) are represented collectively in the study by the level of implementation (reach) and sustainment (re-engagement) support strategies. The scenarios considered in this study examine how changes in the organisational domains interact with the individual domains of reach and maintenance to affect participation rates and the cost of implementation. Cohorts of individual participants are the primary model unit of analysis; as such, the insights derived are focused on the dynamics that arise as these cohorts move through varying levels of participation and engagement.

**Table 1:** Definitions of RE-AIM framework and scope within the system dynamics model

Domain	Definition	How this is reflected in the model
Reach	The absolute number, proportion, and representativeness of individuals who are willing to participate in a given initiative, intervention, or program [6]	**Initial reach**: After learning about the health program, individuals willing to participate immediately begin engaging with the program’s protocol. Higher reach incurs a higher cost due to the additional resources required
		**Delayed reach**: Individuals who do not immediately participate can engage in the program at a later stage. The likelihood of delayed reach depends on how effective supportive strategies are at increasing the willingness to participate. More intensive support strategies incur higher implementation costs
Effectiveness	The impact of an intervention on important outcomes, including potential negative effects, quality of life, and economic outcomes [7]	The impact on health, changes to health care spending, and quality of life occur downstream of participation and were assumed not to differ between scenarios
Adoption	The absolute number, proportion, and representativeness of settings and intervention agents who are willing to initiate a program	Intervention agents are required to enable exposure to the population. Changes to this domain were beyond the focus of this model and do not differ between scenarios
Implementation	*Settings level: *The intervention agents’ fidelity to the elements of an intervention’s protocol, including consistency of delivery, the time required, adaptations made, and the costs of implementation [7]	*Settings level:* Additional supportive strategies to increase participation through increased reach (both initial and delayed) and maintenance (decreasing disengagement and supportive re-engagement) incur higher implementation costs based on how many individuals are unengaged and the intensity of supportive strategies to engage them
	*Individual level: *Clients adhere to the program and implementation strategies [6]	The incremental cost of delivering enhanced reach and maintenance is captured to reflect the increased resources of supportive strategies
		Individual-level aspects of implementation and settings-based adaptations and fidelity are outside the model’s scope
Maintenance	The extent to which behaviour is sustained in a program and to what extent the program or policy becomes institutionalised or part of the routine organisational practices [6, 7]	**Disengagement**: An engaged individual has a likelihood of disengaging from the program. Lower disengagement incurs higher costs due to the additional resources required
	Includes proportion and representativeness of settings that continue the intervention	**Re-engagement**: Individuals who disengage from the program have a chance to re-engage at a later stage. The likelihood of re-engagement depends on how effective supportive strategies are at increasing the willingness to recommence participation. More intensive support strategies incur higher implementation costs

The SD model was initialised with a hypothetical population of 5000 individuals who did not know about the health program. The model was run for 50 simulated years, with equation updates occurring 12 times per year. All simulation modelling was conducted in Stella v1.9.2 (ISEE Systems, Lebanon, NH, USA). Data preparation and analysis were conducted in R v4.4.1. Additional details on the model structure can be found in the supplementary materials.

### Outcomes

#### Reach

Once participants are exposed to the public health program, a proportion of the cohort is willing to participate. Reach is commonly measured as the proportion of the target population participating in the health initiative [6]. This proportion is calculated as the number of individuals participating in the health program divided by the total population.

#### Implementation—cost of delivery

An incremental cost of the program’s delivery was estimated using per-participant activity costs for each factor affecting the flow of individuals through the system. Changes to the flows between model components represent organisational aspirational targets to influence the system. Each targeted rate was given a hypothetical activity cost per person, reflecting the increasing implementation support needed to achieve that target. The relationships between cost and implementation targets were developed to reflect the costly nature of increasing reach and decreasing disengagement via maintenance and sustainment. Achieving an organisational target follows an exponential cost curve, with disengagement following a negative exponential relationship. Increased costs may include increased staff, additional resourcing, and increased reminders or notifications for participants to engage and follow a program’s protocol. The costs are collected for each person; they flow through the corresponding implementation stages and are aggregated to estimate the cumulative cost of implementing the program. As scenarios change to the underlying implementation pathways and the strength of flows between stages, the cost of varying implementation targets and the corresponding population flows through the system result in different total implementation costs. Further details outlining the relationships between program implementation targets and costs can be found in the supplementary material.

### Scenario analysis

Scenario analysis was conducted to explore the interaction between input parameters for reach and disengagement with the model outcome, participation, and cost outcomes. These scenarios examine the implications of enhancing support to provide an understanding of the existing research that advocates for greater reach and sustainability of public health programs [26]. Scenario A assessed the relationship between the outcomes when no support is provided for re-engagement or reach. Scenario B considered a high level of support for re-engagement, resulting in 94% of the disengaged population moving back to participation in the program per model year. Conversely, Scenario C allowed for enhanced reach by increasing the number of individuals who did not initially uptake the program and eventually participated later (delayed reach). Finally, a best-case scenario was used to explore the dynamics when re-engagement and reach strategies were in place. For each of the scenarios, the initial reach and disengagement were sequentially tested between 0 and 100%, allowing for adequate exploration of these parameters across the four scenarios. Table 2 provides a summary of the scenario parameters with corresponding notation and equations used in Fig. 1.

**Table 2:** Scenario input values

			Scenarios			
		Units	A (no support)	C (Enhanced reach)	B (re-engagement support)	D (both strategies supported)
Flow rates	Exposure	Individuals/year	150			
	Proportion initially willing to join the program (initial reach)	Proportion of cohort/year	Sequentially sampled 0 to 1			
	Proportion that disengages from the program		Sequentially sampled 0 to 1			
	Proportion willing to join the program later (delayed reach)		0.00	0.94	0.00	0.94
	Proportion that re-engages in the program		0.00	0.00	0.94	0.94
Stock Initial values	Population unaware of the program	Individuals	5000			
	Population not engaged in the program		0			
	Population not initially participating in the program		0			
	Population participating in the program		0			
	Population disengaged in the program		0			

## Results

**Fig. 2:**
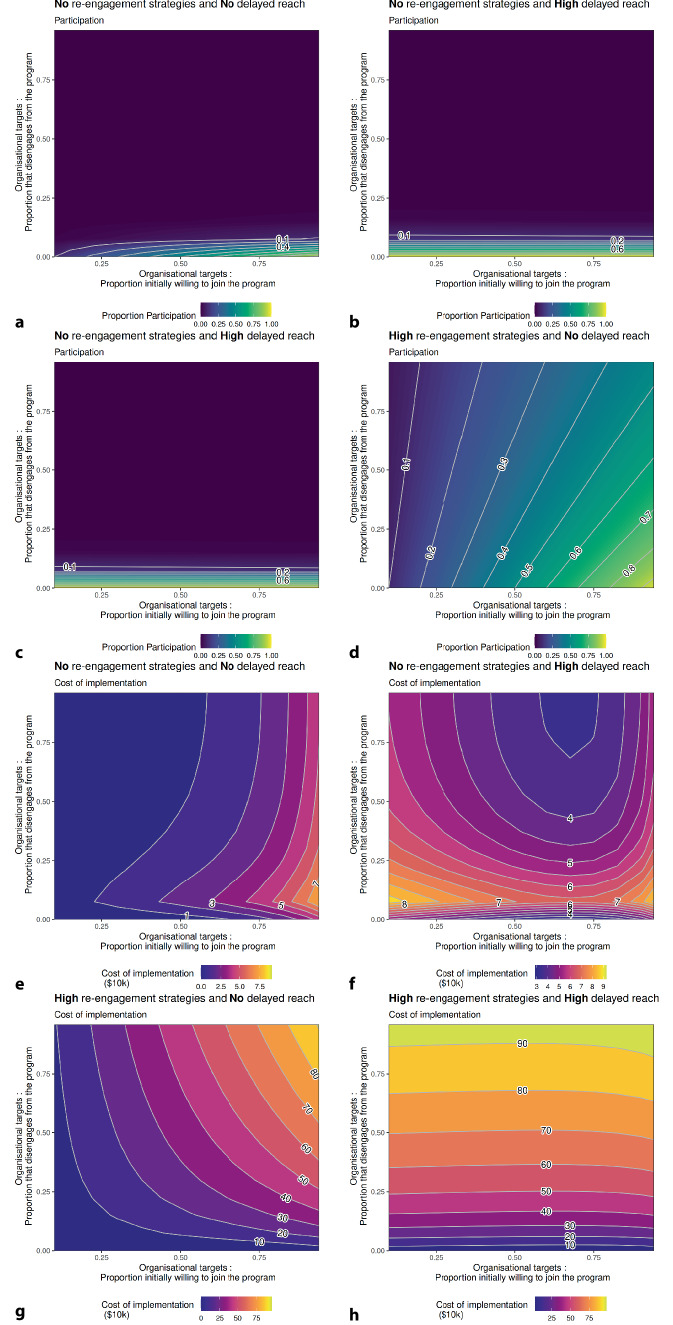
Participation and implementation cost, by disengagement and reach

Figure 2 shows the interaction between reach and disengagement for the primary outcomes: participation and cost across the four scenarios at the end of the simulation. Results are presented for scenario 1, where no support is provided for delayed reach or re-engagement (Fig. 2a, e); scenario 2, which involves enhancing reach by increasing the willingness of participation in those that did not initially uptake the program, i.e., delayed reach (Fig. 2b, f); scenario 3, characterised by high re-engagement (Fig. 2c, g); and Scenario 4, where support is provided for both re-engagement and delayed reach (Fig. 2d, h).

### Scenario 1: No re-engagement and no delayed reach

Low participation was observed in this scenario, with no support for delayed reach or re-engagement (Fig. 2a). High participation was achieved only when the initial reach was high, and disengagement was low. Additionally, the relationship between reach and disengagement is not linear, with disengagement being the dominant factor.

Figure 2e shows the cumulative cost of implementing this scenario while varying the target rate for initial reach and disengagement. These results show that high reach and low disengagement increase the cost of implementation. However, since the alternative pathways (re-engagement and delayed reach) are not supported and, therefore, not utilised, the system does not incur the cost of these additional support strategies, resulting in a lower overall cost for this scenario compared to other scenarios tested.

### Scenario 2: No re-engagement and high delayed reach

Low participation was also observed when enhanced delayed-reach strategies were enacted (Fig. 2b). Disengagement remained the dominant factor, as alternative pathways to participation (delayed reach) were utilised, reducing the reliance on high initial reach.

Unlike scenario 1, the cost profile for this scenario (Fig. 2f) showed that increased costs occurred when the initial reach was low. Two mechanisms led to higher costs: (1) High initial reach is costly to implement, and (2) when initial reach is low, the system requires additional support through reach strategies, which can be costly to sustain.

### Scenario 3: High re-engagement and no delayed reach

Support for high re-engagement (Fig. 2c) resulted in a strong two-way interaction between initial reach and disengagement. Intuitively, the greatest participation occurred for high initial reach and low disengagement. However, disengagement was no longer a dominant factor, where moderate to high reach was obtainable even when these implementation characteristics were not at their respective maximums.

The cost of implementing this scenario (Fig. 2g) can increase rapidly when the population is willing to participate (high reach), coupled with high disengagement and re-engagement. This combination creates a cycle in which participants bounce between engagement and disengagement. This cycle drives up implementation costs as more individuals utilise support strategies that incur an activity cost.

### Scenario 4: High re-engagement and high delayed reach

The results of this best-case scenario (Fig. 2d) show the most combinations that provided the highest participation in the program. These results reflected scenario 2 and 3 findings, where support for enhanced delayed reach reduces the impact of low initial reach, and disengagement is no longer a dominant factor.

Like scenario 3, highly utilised re-engagement strategies allow the population to move between engagement and disengagement, resulting in higher implementation costs (Fig. 2h). The cost profile for this scenario was driven even higher by the increased cost of delayed reach, resulting in the highest cost of all tested scenarios.

Overall, scenarios with enhanced delayed-reach support resulted in greater utilisation of alternative pathways to participation, thus removing the dependency on initial reach. Furthermore, on average, scenarios with higher re-engagement resulted in greater participation, as those who disengaged were more able to re-engage in the program later. High utilisation of these alternative support pathways for participation increased the overall cost of delivering the program because participants could cycle between engagement and disengagement.

## Discussion

Modelling hypothetical systems can be useful to elucidate distinct pathways that drive system outcomes and assess the validity of proposed pathways [14]. The model structure presented in this research focused on the reach, maintenance, and cost of implementation components of the RE-AIM framework. The purpose of the proof-of-concept model presented in this research was to enable specific system dynamics modelling applications to incorporate RE-AIM in policy analysis. The scenario analysis conducted in this research examines the dynamics of enhanced reach and maintenance strategies on active participation and cost. The insights from this research explore these concepts and provide an understanding of the usefulness of the proposed pathways. Through these aims, this research demonstrates how dynamic modelling concepts can be used to explore the interactions between different strategies and their impact on both engagement and cost, assisting in program planning.

The insights from the modelled scenarios reinforce the complex nature of implementation science and illustrate the non-linear dynamic relationships that can influence our understanding of the system [27, 28]. While enhancing reach strategies can help engage those who do not immediately uptake a program, the scenario shows that disengagement was a more dominant influence in maintaining active participation, affirming the need for focused research on the sustainability of prevention programs [29]. Strategies with higher support for delayed reach and re-engagement resulted in increased costs. These costs arose from the utilisation of alternative pathways to participation and the cycle between participation and non-participation, which incur a cost at each stage. Previous simulation modelling research indicated similar findings: Although increasing resources for program maintenance led to a higher overall cost, it also improved program delivery, reduced effectiveness erosion, and sustained a higher steady state of adoption [30]. This study’s findings demonstrate the additional resources required to implement enhanced strategies and emphasise the need to develop cost-effective methods to lower delivery costs and improve overall acceptability [31].

Researchers and program evaluators have highlighted the importance of sustainability of public health initiatives [11, 32–34]. This study further emphasises this sentiment but illustrates the high-cost implications of re-engagement on maintaining reach and engagement (i.e., maintained behaviour) with the intended population. Conversely, identifying effective and cost-effective sustainability strategies that can maintain engagement and reduce the need for re-engagement are significant for enhancing impact and reducing cost [31]. These results thus emphasise the importance of identifying practical and cost-effective sustainment strategies that can reduce disengagement while supporting maintained engagement, consistent with current sustainability recommendations [26, 29]. This has implications for decision-makers and program planning, highlighting that a deep understanding of implementation characteristics, such as the cost associated with supporting engagement, is necessary to allocate scarce public resources and plan effectively [7, 35].

### Limitations

The model structure and data presented in this paper are hypothetical; the intent was to produce generalised implementation insights without specific application, to explore RE-AIM, and to provide a proof of concept for future modelling applications. However, as shown in the results, complex dynamics influence the resulting insights, which may not hold true for all applications or combinations of input parameters. Furthermore, we externalised some organisational domains of RE-AIM (i.e., adoption, implementation, and maintenance), focusing only on variations in reach and sustainment support through these scenarios. Greater details may be needed to differentiate between these organisational components and show how their intensity and cost may differ. However, this was beyond the scope of this study. We also assumed that the public health program has evidence of effectiveness, which did not change over time or across different populations and settings.

System dynamics modelling is a cohort-level analysis, assessing changes in participation states as cohorts of individuals [16]. This method implies that individuals’ future movements are not influenced by their previous experiences [19]. This characteristic of SD modelling may be particularly impactful when there is a high level of support for re-engagement. Participants’ re-engagement rates in a public health program would likely decrease for every subsequent re-engagement loop. Without the ability to track individuals, this model assumes that every disengaged individual has the same likelihood of re-engaging, irrespective of their previous experience in the program.

Future extensions of this SD model should incorporate the added complexities of nesting individual domains of RE-AIM within organisational domains of RE-AIM. This would allow for broader model scope to consider issues such as equity and changes in the evidence base of the public health program [7]. Furthermore, specific applications contextualised in a grounded population may emphasise the further usefulness of SD modelling as a planning tool within implementation research.

## Conclusion

This study focused on qualitative insights regarding support strategies for re-engagement and alternative pathways to increase reach for a generalised public health initiative. Multiple complex characteristics interact when considering and applying the RE-AIM framework in public health programs. This study highlights the importance of strategies to help establish and maintain engagement with the population. However, the high cost of these supportive strategies stresses the need for innovative and efficient ways of implementing these support strategies. System dynamics modelling and simulation studies can support implementation planning by considering the trade-offs between reach and disengagement and their impact on outcomes, helping to develop realistic goals for public health programs. Future research can further test specific applications of this modelling approach to support planning and explore the nature of implementation characteristics in differing settings.

### Model availability.

The model is available at https://exchange.iseesystems.com/models/player/simon-chiu/enhancing-noncommunicable-public-health-programs-a-system-dynamics-approach-to-understanding-reach-maintenance-and-implementation-costs

## Supplementary Information

This supplemental material provides additional data, figures and equations that support the findings presented in the main manuscript.

## Declarations

This study involves the analysis of hypothetical data and did not require an ethics review.
