# Supplementary material for: Enhancing noncommunicable public health programs: A system dynamics approach to understanding reach, maintenance, and implementation costs
Source: Bundesgesundheitsblatt Gesundheitsforschung Gesundheitsschutz. 2025 May 30;68(7):769–77. doi: 10.1007/s00103-025-04069-7 (PMC12254156; doi:10.1007/s00103-025-04069-7)
Supplement: Supplementary file 1 — This supplemental material provides additional data, figures and equations that support the findings presented in the main manuscript. [file 103_2025_4069_MOESM1_ESM.pdf]

Supplementary Materials: Enhancing Noncommunicable Public Health Programs: A System Dynamics Approach to Understanding Reach, Maintenance, and Implementation Costs

Implementation model

Structure

Figure S1 illustrates the modelled process by which the population progresses during the implementation of the health program. Initially, before the program's rollout, the population is unaware of its existence. As awareness grows through exposure, individuals become informed about the program. Based on model inputs, a portion of the population will choose to participate initially. In contrast, others may remain unengaged, possibly becoming involved later through a delayed reach pathway. Participants who actively engage in the program may subsequently disengage. The model also accounts for pathways that enable re-engagement.

The equations used in the model are presented in Table S1.

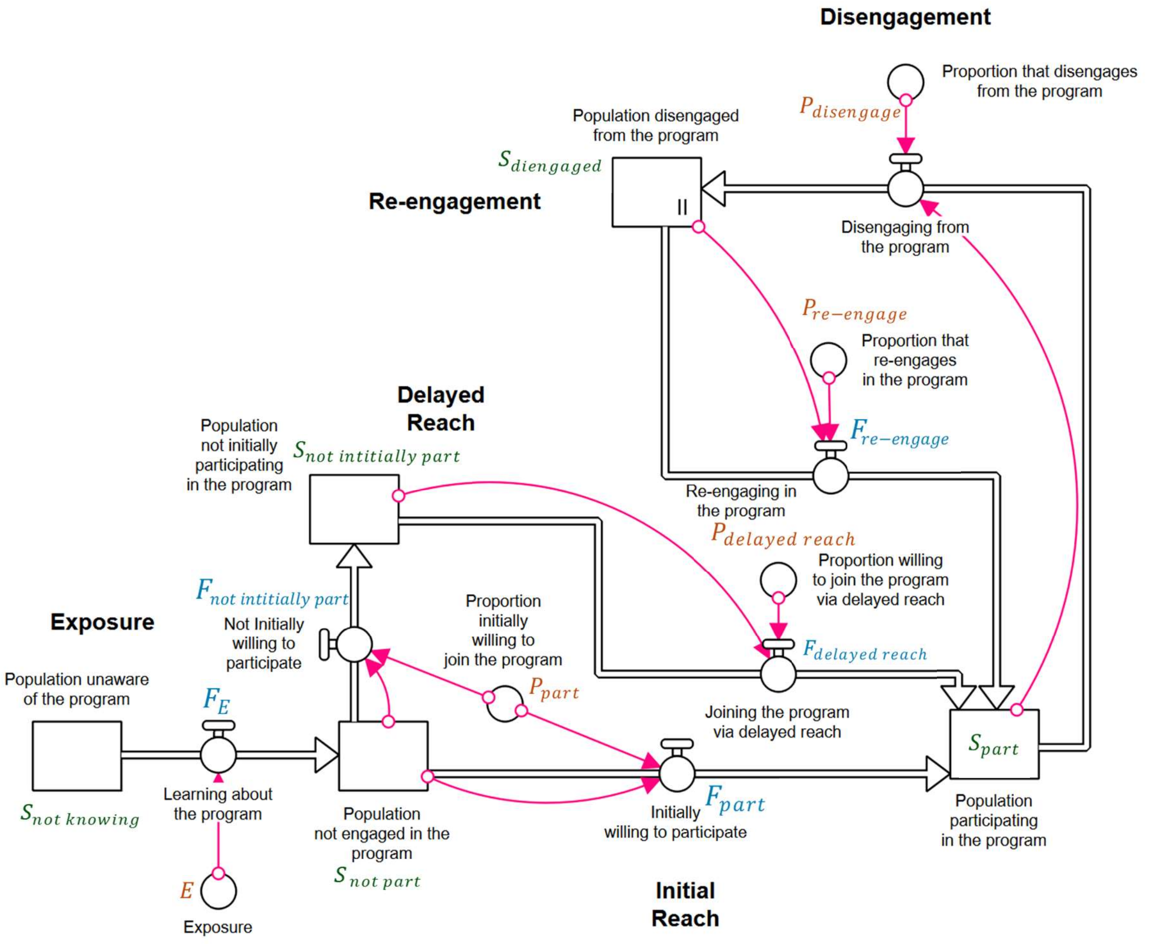

Equations

Table S 1 Equations for implementation structure

Notation	Description	Equation
F_E	The number of individuals that learn about the program per model year.	$F_E = E$
F_{reach}	The number of individuals initially willing to join the program per model year.	$F_{reach} = S_{not\ part} \times P_{reach}$
$F_{disengage}$	The number of individuals that disengage in the program per model year.	$F_{disengage} = S_{part} \times P_{disengage}$
$F_{not\ititally\ part}$	The number of individuals who ***do not*** initially to participate in the program per model year.	$F_{not\ intiall\ part} = S_{not\ part} \times (1 - P_{reach})$
$F_{delayed\ reach}$	The number of individuals who choose to participate in the program ***after not initially choosing to participate*** via delayed reach per model year.	$F_{tert\ reach} = S_{not\ initially\ part} \times P_{tert\ reach}$
$F_{re-engage}$	The number of individuals who re-engage in the program per model year.	$F_{re-engag} = S_{disengaged} \times P_{re-engage}$
$S_{t,not\ knowing}$	Population not knowing about program at time (t)	$S_{t,not\ knowing} = S_{t0,not\ knowing} + \int_{t0}^{t} (-F_E)\ dt$
$S_{t,not\ part}$	Population not engaged in the program at time (t).	$S_{t,not\ part} = S_{t0,not\ part} + \int_{t0}^{t} (F_E - F_{reach} - F_{not\ ititally\ part})\ dt$
$S_{t,part}$	Population participating in the program at time (t).	$S_{t,\ part} = S_{t0,part} + \int_{t0}^{t} (F_{reach} + F_{tert\ reach} + F_{re-engage} - F_{disengage})\ dt$
$S_{t,not\ ititally\ part}$	Population who did not initially participate at time (t).	$S_{t,not\ ititally\ part} = S_{t0,ititally\ part} + \int_{t0}^{t} (F_{not\ ititally\ part} - F_{tert\ reach})\ dt$
$S_{disengage}$	Population disengaged from the program at time (t).	$S_{t,disengaged} = S_{t0,disengaged} + \int_{t0}^{t} (F_{disengage} - F_{re-engage})\ dt$
**Outcome**	***Proportion of population engaged*** : the number of individuals participating in the health program divided by the total population.	$\frac{S_{t,part}}{(S_{t,not\ knowing} + S_{t,not\ part} + S_{t,part} + S_{t,not\ ititally\ part} + S_{t,disengage})}$

Cost of delivery

Structure

Figure S2 shows the model structure used to calculate the cost of delivery. This structure employs a series of stocks and flows to collect the activity costs for each RE-AIM domain represented in the model. Each stock starts at zero cost and increases based on the number of individuals flowing through each model component. These flows are multiplied by the associated per-person activity cost. Since there are no outflows, these stocks accumulate each activity costs, which are then summed to estimate the total delivery cost.

The changing per-person cost of achieving an organisational target for each domain was assumed to follow an exponential relationship, reflecting the increased resources needed to achieve a favourable target. Figure S3 presents these relationships. A less favourable target (e.g., low reach, high disengagement) approaches zero cost, reflecting an environment where no resources are allocated to implementing performance strategies. As these targets approach favourable levels (e.g., high reach, low disengagement), nearing 100%, the costs increase, reflecting the additional resources required to achieve these targets.

Figure S 2 Model structure to estimate the total cumulative cost of delivery with model equation notation

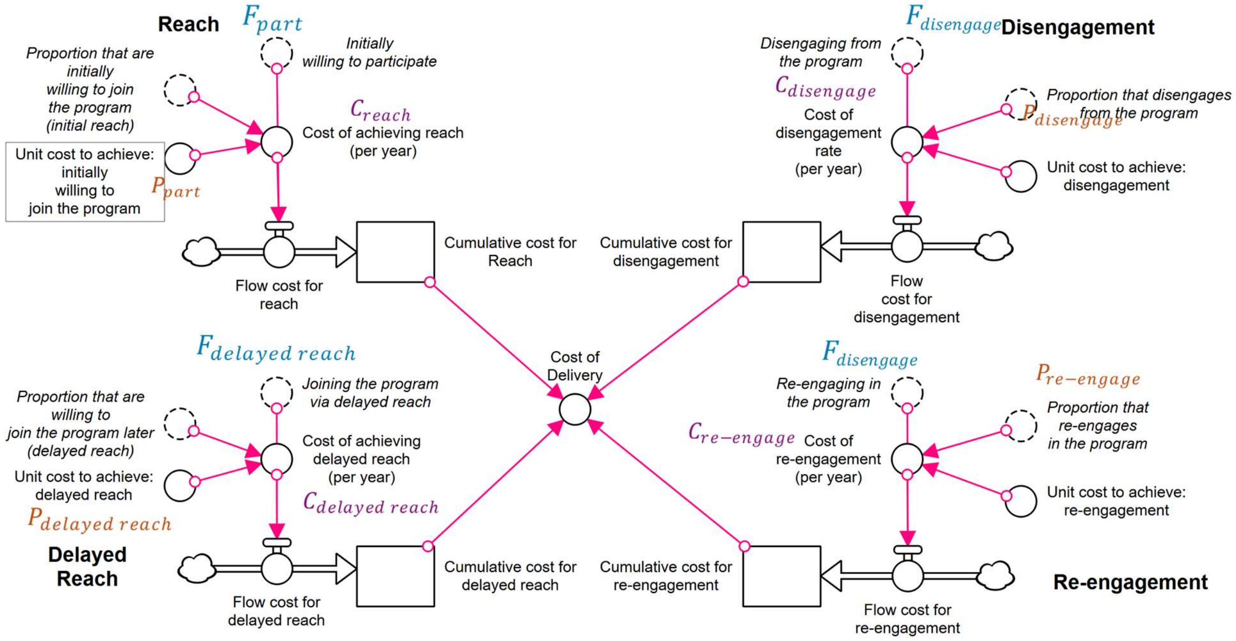

Figure S 3 Cost profile of organisational aspirational for reach, disengagement, re-engagement and delayed reach

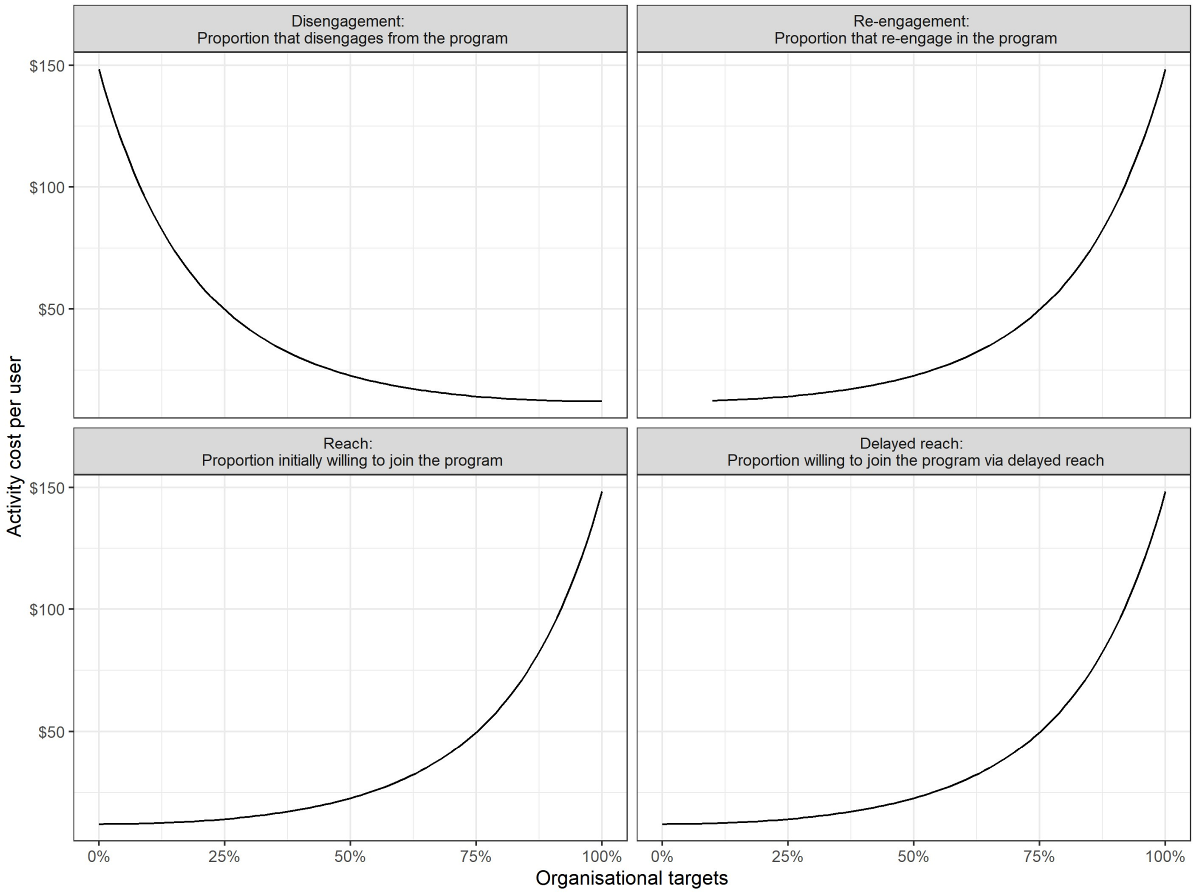

Equations

Table S 2 Cost of delivery equations

Notation	Description	Equation
C_{reach}	Hypothetical per-person cost of achieving organisational reach target.	$e^{2.5(1+P_{part}{}^{2})}$
$C_{disengage}$	Hypothetical per-person cost of achieving organisational disengagement target.	$e^{2.5(1+(1-P_{disengage})^{2})}$
$C_{delayed\ reach}$	Hypothetical per-person cost of achieving organisational delayed-reach target.	$e^{2.5(1+P_{tert\ reach}{}^{2})}$
$C_{re-engag}$	Hypothetical per-person cost of achieving organisational re-engagement target	$e^{2.5(1+P_{re-engag}\ {}^{2})}$
**Outcome**	***Total cumulative cost of delivery*** : the cumulative system flow multiplied by the per-person cost for each components , summed together.	$\sum_{t=0}^{t} F_{part}\, C_{reach} + \sum_{t=0}^{t} F_{disengage}\, C_{disengage} + \sum_{t=0}^{t} F_{delayed\ reach}\, C_{delayed\ reach} + \sum_{t=0}^{t} F_{Re-engage}\, C_{Re-engage}$
